# Differences in Gene Expression of Pear Selections Showing Leaf Curling or Leaf Reddening Symptoms Due to Pear Decline Phytoplasma

**DOI:** 10.3390/plants11030427

**Published:** 2022-02-04

**Authors:** Mina Kaviani, Paul H. Goodwin, David M. Hunter

**Affiliations:** 1 Department of Plant Agriculture, University of Guelph, Guelph, ON N1G 2W1, Canada; davidmhunter13@gmail.com; 2 School of Environmental Sciences, University of Guelph, Guelph, ON N1G 2W1, Canada; pgoodwin@uoguelph.ca

**Keywords:** pear decline, phytoplasma, gene expression, foliar symptoms, phloem

## Abstract

While host gene expression has been related to symptoms associated with different phytoplasma diseases, it is unknown why some phytoplasmas are associated with different symptoms in genotypes of the same plant species. Pear tree selections showed symptoms of either leaf reddening (selection 8824-1) or leaf curling (selection 9328-1) associated with pear decline (PD) phytoplasma presence. PD populations were similar in leaves and shoots of the two selections, but in the roots, populations were significantly lower in selection 8824-1 than in 9328-1, indicating greater resistance. For host carbohydrate metabolism gene expression in PD-infected tissues, significant up-regulation in selection 8824-1 was observed for a sucrose synthase gene in leaves and an acid invertase gene in leaves and roots. These features have been associated with localized higher sugar levels in phytoplasma-infected tissues, and thus may be related to leaf reddening. For host stress/defense response gene expression in PD-infected tissues, significant up-regulation of a phenylalanine ammonia lyase gene was observed in PD-infected shoots of both selections; however, up-regulation of alcohol dehydrogenase gene in shoots, a chitinase gene in all tissues and a phloem protein 2 gene in roots was only observed for selection 8824-1. These changes indicate greater triggered innate immunity in roots associated with lower PD populations and leaf reddening. Leaf reddening may be related to changes in gene expression associated with increased sugar levels in leaves and stronger immune responses in several tissues, while leaf curling may be due to water stress resulting from dysfunctional root associated with higher PD populations in the roots.

## 1. Introduction

Pear decline (PD) disease of pear (*Pyrus communis* L.) is associated with the presence of *Candidatus* Phytoplasma pyri, a member of the apple proliferation group (16SrX group), that causes a slow death of pear trees on *P. calleryana* and *P. communis* rootstocks. PD symptoms include poor fruit development, reduced shoot growth, phloem necrosis, leaf curling and premature leaf reddening [1]. Leaf curling begins in late summer and is most noticeable in the fall, when the midrib curls downward resulting in the leaf tip sometimes touching the ventral side of the midrib or petiole, thus forcing the leaf blades to curl upwards. Curled leaves may become thickened and acquire a deep red purplish hue. In early fall, premature foliar reddening contrasts with the leaf yellowing typical of normal leaf senescence on healthy trees [2]. Within *P. communis,* some cultivars, such as Bartlett, Old Home and Beurre Hardy, show PD-induced leaf curl symptoms with little leaf reddening, while other cultivars, such as Comice, show leaf reddening with only slight leaf curling, indicating that there is a host genotype effect on foliar symptoms [3,4].

Trees of several pear breeding selections grown on *P. communis* rootstocks in the greenhouses at the Agriculture and Agri-Food Canada (AAFC) Vineland Station, Ontario research station have tested positive for PD phytoplasma presence by PCR [5]. PD-infected trees of selection 8824-1 have consistently shown only leaf reddening, while PD-infected trees of selection 9328-1 have consistently shown only leaf curling each August and September over six years (Hunter, D, unpublished data). As trees of these lines were grown adjacent to each other, different foliar symptoms are unlikely to be due to environmental effects.

Symptoms of several phytoplasma diseases have been associated with the population sizes of the phytoplasma inside the plant. For example, the severity of leaf yellowing and leaf curling associated with by the European stone fruit yellows phytoplasma in *Prunus salicina*, *P. armeniaca*, *P. persica* and *P. tomentosa* was positively correlated with higher titres of Ca. P. prunorum during the growing season [6]. Roggia et al. (2014) reported a similar positive correlation between flavescence dorée (FD) phytoplasma titre in the spring and the severity of foliar symptoms (yellowing and downward leaf curl) in *Vitis vinfera* cvs. Barbera and Nebbiolo [7]. Symptomatology of some phytoplasma diseases has also been related to how the host is affected by the infection. FD and Bois Noir (BN) phytoplasmas cause discoloration and downward rolling of leaves in grapevines, which was related to increased expression of carbohydrate-related metabolism genes, such as acid invertase (AIV) and sucrose synthase (*SUSY*) [8]. Increased acid invertase and sucrose synthase gene expression simultaneously decreases phloem loading and increases phloem unloading in source leaves as they switch from carbohydrate sources to sinks [9]. As a result, phytoplasma infection reduces sugar transport in the phloem, leading to carbohydrate accumulation in leaves with consequent leaf yellowing and premature senescence and abscission of the leaves [10].

Symptomology might also be related to changes in host stress/defence responses. Increased expression of genes for defence-related proteins, such as alcohol dehydrogenase (*ADH*), class III chitinase (*CHIT.C3*) and phenylalanine ammonia lyase (*PAL*), occurred during discoloration and downward rolling of grape leaves following infection by the FD and BN phytoplasmas [11]. Up-regulation of phloem protein 2 (PP2) in the phloem of apple proliferation (AP)-infected apple was linked with leaf discoloration [12]. Alcohol dehydrogenase is involved in alcohol utilization under hypoxic conditions, which occur during phytoplasma infection [10], class III chitinase can digest cell walls of bacteria that would likely not directly affect phytoplasmas as they lack cell walls [13,14,15], PAL catalyzes the first step of the phenylpropanoid pathway to synthesize many antimicrobial defence compounds and increased PAL activity has been associated with increased phenolic compounds in the phloem [16,17,18,19,20,21], and phloem proteins (*PP*) 1 and 2 physically limit pathogen spread by plugging sieve pores in the phloem as well as synthesizing and modulating systemic signals, such as cytosolic Ca^2+^, contributing to innate immunity [12].

To gain insights into the different PD foliar symptoms in selections 8824-1 and 9328-1, the levels of PD phytoplasma in leaves, shoots and roots of non-infected and PD-infected trees of the two pear selections were compared, and expressions of genes involved in carbohydrate metabolism and defence mechanisms were determined.

## 2. Results

### 2.1. Foliar Symptoms in Selections 8824-1 and 9328-1

**Figure 1:**
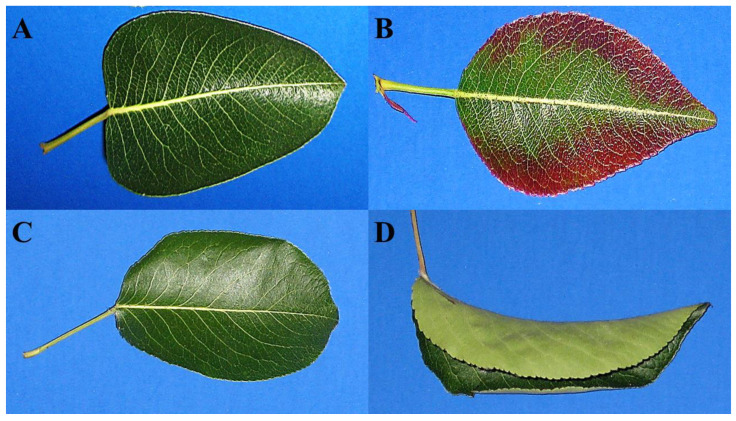
Leaves of *Pyrus communis* selections 8824-1 and 9328-1 without and with PD phytoplasma foliar symptoms. (**A**) A non-symptomatic leaf in selection 8824-1, (**B**) a leaf with reddening symptoms in selection 8824-1, (**C**) a non-symptomatic leaf of selection 9328-1 and (**D**) a leaf with curling symptom in selection 9328-1. Images are of mature leaves from the middle part of the tree (approx. 150 cm height).

Mature leaves from clonally-propagated non-symptomatic trees of selections 8824-1 and 9328-1 were dark green and flattened (Figure 1A,C). For PD-symptomatic trees of selection 8824-1, however, approx. 75–80% of the mature leaves, but none of the immature leaves, showed red margins starting in mid-August becoming more severe by the end of September (Figure 1B). For PD-symptomatic trees of selection 9328-1, leaf curling was first observed in approx. 80–90% of mature leaves in mid-August and then developed in all mature leaves around mid-September (Figure 1D). These symptoms occurred consistently over the two growing seasons of study.

The source of phytoplasma infection for the PD-symptomatic trees is unknown, but the buds used for propagating these trees in 2006 came from the original seedling trees which were grown in a field evaluation orchard at the Jordan Farm of AAFC-Vineland since 2000. PD-infection of some of the original seedling trees of selections 8824-1 and 9328-1 most likely occurred by natural spread vectored by pear *psylla* (*Cacopsylla pyri*) Selections 8824-1 and 9328-1, which share the common ancestors of Harrow Delight, Bartlett, Old Home and Purdue 80-51, but both selections have open pollinated backgrounds meaning that the pollen sources of those pear progenitors are unknown (Figures S1 and S2). Although selection 9328-1 comes from a much more diverse background than selection 8824-1, these selections appear to have both PD-tolerant (Old Home) [3] and PD-susceptible (Max Red Bartlett) [22] progenitors. The appearance of non-symptomatic trees of selections 8824-1 and 9328-1 is relatively similar, except for the higher level of red color in mature fruit of selection 8824-1 than in selection 9328-1.

### 2.2. Detection and Quantification of PD Phytoplasma in Selections 9328-1 and 8824-1

**Figure 2:**
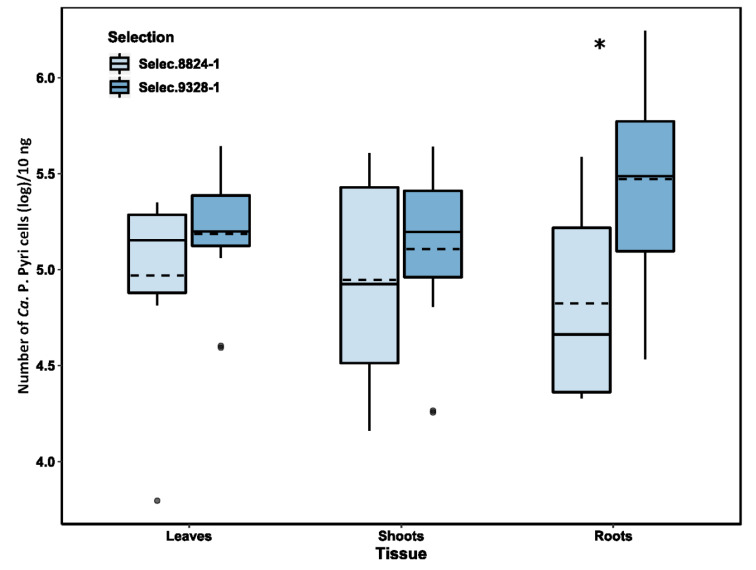
Box plots representing the relative number of pear decline phytoplasma in 10 ng/µL of DNA extracted from leaves, shoots and roots (8 samples per tissue for each selection) of *Pyrus communis* selections 8824-1 and 9328-1 as measured by TaqMan quantitative real-time PCR (qPCR) analysis with a PD-specific primer pair (PHYTOF33/PHYTOR3) (Table 1). Each box represents the interquartile range containing 50% of the values. The whisker lines indicate the range from the highest and lowest values, excluding outliers. Within the boxes, the dashed lines indicate the means and solid lines indicate the medians. Asterisks indicate the means that were significantly different (Tukey’s post hoc test, *p* < 0.05).

PD was not detected in non-symptomatic plants. For PD-positive plants, the average number of PD phytoplasma was not significantly different between leaves and shoots within each of selection, although the variation was greater in shoots than in leaves (Figure 2). There were also no significant differences in PD phytoplasma numbers for leaves and shoots between the two selections, but for roots, PD phytoplasma numbers in selection 9328-1 were significantly higher than in leaves and shoots of selection 9328-1 as well as significantly higher than in roots of selection 8824-1.

### 2.3. Expression of Genes Involved in Carbohydrate Metabolism

**Figure 3:**
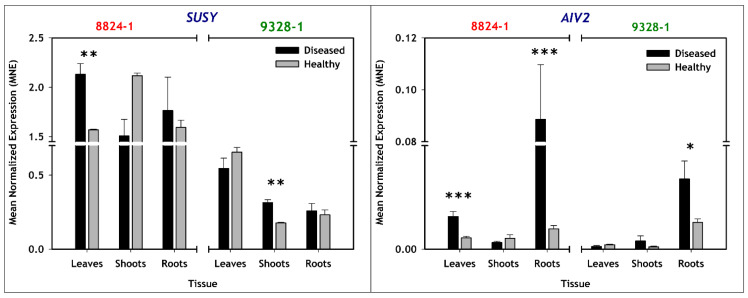
Bar plots representing relative expression of genes involved in carbohydrate metabolism (*SUSY* and *AIV2*) in leaves, shoots and roots of non-infected (grey) and PD-infected (black) pear selections 8824-1 and 9328-1. The data are expressed as mean normalized expression (MNE) against *GAPDH*, which is proportional to the relative quantity of mRNA in a given tissue. The MNE of each sample was determined from 3 biological replications with 2 repeats per biological replication. Asterisks indicate means that were significantly different (Tukey’s post hoc test, *p* < 0.05) with standard errors shown, and *, **, and *** indicating statistical significances of *p* < 0.05, *p* < 0.01, and *p* < 0.001, respectively.

A comparison of non-infected and PD-infected tissues of selection 8824-1 revealed significantly higher *SUSY* expression only in PD-infected leaves, whereas for selection 9328-1, *SUSY* expression was only significantly higher in PD-infected shoots (Table 2, Figure 3). For non-infected tissues, selection 8824-1 had higher basal levels of *SUSY* expression than selection 9328-1, with an average of 1.75 MNE (Mean Normalized Expression, see Materials and Methods for calculation method) for selection 8824-1 tissue types compared to an average of 0.36 MNE for all selection 9328-1 tissue types. For *AIV2* expression, comparisons of non-infected and PD-infected tissues showed significantly higher expression only in PD-infected leaves and roots for selection 8824-1, while for selection 9328-1, there was significantly higher expression only in PD-infected roots (Table 2, Figure 3). Unlike *SUSY* expression, basal levels of *AIV2* expression in non-infected tissues were similar between the two selections for all the tissue types.

### 2.4. Expression of Stress/Defense Related Genes

**Figure 4:**
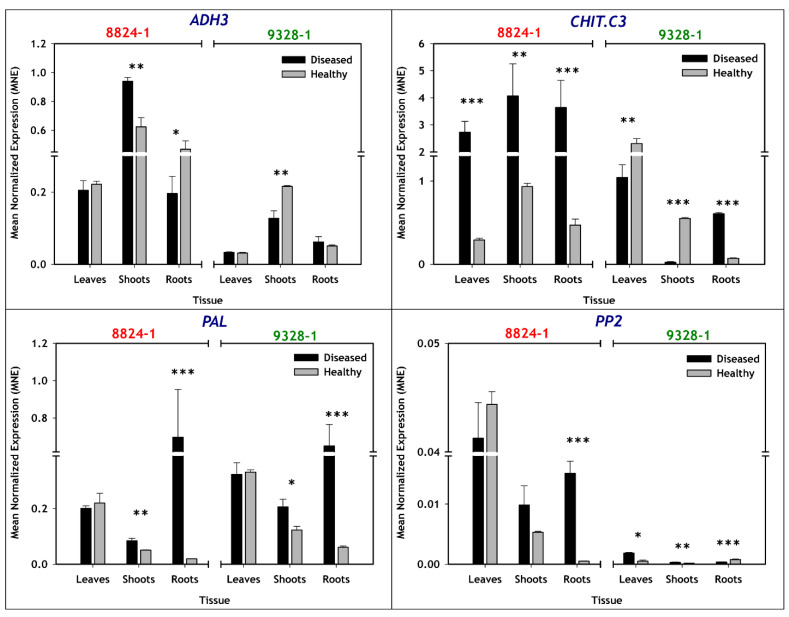
Bar plots representing relative expression of stress/defense-related genes (*ADH3*, *CHIT.c3*, *PAL* and *PP2*) in leaves, shoots and roots of non-infected (grey) and PD-infected (black) pear selections 8824-1 and 9328-1. The data are expressed as mean normalized expression (MNE) against *GAPDH*, which is proportional to the relative quantity of mRNA in a given tissue. The MNE of each sample was determined from 3 biological replications with 2 repeats per biological replication. Asterisks indicate means that were significantly different (Tukey’s post hoc test, *p* < 0.05) with standard errors shown, and *, **, and *** indicating statistical significances of *p* < 0.05, *p* < 0.01, and *p* < 0.001, respectively.

Comparing non-infected and PD-infected tissues of selection 8824-1, *ADH3* expression was significantly higher only in PD-infected shoots and significantly lower only in PD-infected roots (Table 2, Figure 4). For selection 9328-1, the only significant difference between non-infected and PD-infected tissues was the lower *ADH3* expression in PD-infected shoots. Selection 8824-1 had a higher basal expression level of *ADH3* than selection 9328-1.

*CHIT.c3* (Table 2) expression in selection 8824-1 was significantly higher in all PD-infected tissue types compared to non-infected tissues. However, *CHIT.c3* expression in selection 9328-1 was significantly lower in PD-infected leaves and shoots, and significantly higher in PD-infected roots, compared to the non-infected tissues (Figure 4). Basal levels of *CHIT.c3* expression in all tissue types of non-infected tissues were similar between the two selections. For both selections (8824-1 and 9328-1), *PAL* expression levels were significantly higher only in PD-infected shoots and roots (Table 2, Figure 4). This was the only case where both selections had similar changes in gene expression in response to host PD-infection. Basal levels of *PAL* expression in non-infected tissues were also similar between the two selections in all the tissue types.

For *PP2* expression, a comparison between non-infected and PD-infected tissues showed significantly higher expression only in roots of PD-infected selection 8824-1 and only in leaves and shoots of PD-infected selection 9328-1 (Table 2, Figure 4). In addition, roots of PD-infected selection 9328-1 also showed significantly lower expression compared to non-infected roots (Figure 4). Similar to *ADH3* expression, basal expression of *PP2* was higher in selection 8824-1 than selection 9328-1, but in this case, only for non-infected leaves with 0.045 MNE for selection 8824-1 compared to <0.001 MNE for selection 9328-1.

## 3. Discussion

The most common foliar symptoms produced by phytoplasmas are yellowing, reddening and rolling [23]. For example, leaf yellowing is induced by palm lethal yellowing (LY), rice yellow dwarf, BN and peach X diseases, while leaf reddening is caused by PD, AP, and maize bushy stunt (MBS) diseases, and leaf curling results from peach yellow leaf roll, PD and grapevine yellows diseases [24]. However, it is not known what changes in the host results in these different symptoms. While typically foliar symptoms are specific to a particular phytoplasma disease, PD appears to induce both leaf reddening and curling depending upon the host genotype [3].

Leaf reddening has been linked to the accumulation of anthocyanins and sugars during senescence [25]. However, premature leaf reddening can also be induced by environmental stresses (e.g., drought, high temperature and low light [26]), as well as by pathogen attack (e.g., leafroll virus of grapevines [8]). Leaf curling is also associated with stresses, such as drought [27], ozone [28] and pathogen attack (e.g., tomato yellow leaf curl virus [29]).

One possibility is that the type of foliar symptoms induced by PD was related to the phytoplasma population size in each selection. While PD phytoplasma populations did not differ in the leaves or shoots, there was significantly more PD phytoplasma in the roots of selection 9328-1 than 8824-1. Higher PD phytoplasma populations in roots could cause greater phloem tissue damage resulting in a depletion of sugars for root metabolism, and reduced total root function would then indirectly restrict water and nutrient transport [9]. Thus, the larger population of PD phytoplasma in roots of selection 9328-1 may have caused greater drought stress than in selection 8824-1, resulting in leaf curling in selection 9328-1.

Among the pear genes involved in carbohydrate metabolism examined in this study, significant up-regulated expression of both a sucrose synthase and an acid invertase gene was observed in PD-infected leaves of selection 8824-1, even though the PD population was similar to that of selection 9328-1 in leaves. Sucrose synthase cleaves sucrose to fructose and UDP-glucose, while invertase cleaves sucrose to fructose and glucose [30]. The pear sucrose synthase and acid invertase genes selected in this study were chosen because their orthologs were up-regulated in grapevine leaves infected with FD, BN and Stolbur phytoplasmas [8,9], and periwinkle and tomato leaves infected by Stolbur phytoplasma [31]. Localized increases in sucrose synthase and vacuolar invertase could result in decreased sucrose and increased fructose and glucose levels [32]. Phytoplasmas need to increase host sucrose synthase and vacuolar invertase gene expression because, unlike other types of biotrophic pathogens, a survey of the genomes of over 4 phytoplasmas genomes, including AP phytoplasma that is closely related to PD, showed that they all lack the enzymes in phosphotransferase systems, which is necessary for importing and phosphorylating sugars, such as sucrose, glucose and fructose [10,33,34]. Thus, increasing sucrose synthase and vacuolar invertase activities in host cells will allow for greater breakdown of host sucrose into fructose and glucose for phytoplasma growth [10,35,36].

In addition to supplying the pathogen with utilizable sugars, hexose accumulation can trigger leaf senescence, and hexose-dependent signals can induce a reduction in leaf chlorophyll content and hence photosynthetic activity [37]. For maize bushy stunt (MBS) phytoplasma, which induces both foliar chlorosis and leaf reddening, a reduction in chlorophyll content and accelerated leaf senescence was linked to increased soluble sugars in maize leaves [38]. The leaf reddening in selection 8824-1 could be due to premature leaf senescence arising from a localized accumulation of sugars associated with the up-regulation of sucrose synthase and vacuolar invertase expression in leaves. In contrast, selection 9328-1 may not have shown leaf reddening due to premature leaf senescence as those genes were not up-regulated in leaves with PD infection, but rather were up-regulated only in other tissues.

Among the pear genes involved in stress/defence-related genes examined, expression of a pear ADH-P class alcohol dehydrogenase was examined. ADH-Ps are ethanol-active alcohol dehydrogenases that reduce acetaldehyde to ethanol in anaerobic glycolysis, generating NAD^+^ and ATP when normal respiration is disrupted [39]. Following infection of grapevine by BN and FD phytoplasmas, an ADH-P class alcohol dehydrogenase gene was up-regulated, possibly triggered by intensified hypoxic conditions in phloem tissues resulting from the inhibition of photosynthesis and a subsequent switch to fermentative metabolism with alcohol dehydrogenase generating NAD^+^ and ATP [8,10]. In the current study, the pear ortholog of that alcohol dehydrogenase gene was significantly up-regulated by PD infection for shoots of selection 8824-1 and down-regulated for roots of selection 8824-1 and shoots of selection 9328-1. The increase in expression in PD-infected shoots of selection 8824-1 suggests that this tissue was subjected to the greater hypoxic conditions, which could result in leaf reddening, as has been reported in Arabidopsis [40]. In addition, increased expression of a grapevine ADH-P class alcohol dehydrogenase was linked to anthocyanin accumulation in red grape berries undergoing drying, when the metabolism became anaerobic [41].

Expression of two other defence-related genes, a class III chitinase and a PAL, were also examined. Class III chitinases display substantial lysozyme activity with a probable direct anti-bacterial action [13], and PAL activity results in the eventual production of a wide range of phenolic compounds, such as flavonoids, anthocyanins and lignins, all of which have anti-microbial activity [42]. A class III chitinase gene was up-regulated in maize leaves infected by MBS phytoplasma [15] and grapevine leaves infected by BN phytoplasma [11], and a PAL gene was up-regulated in grapevine leaves infected with Ca. *P. solani* [43] and tomato leaves infected Ca. *P. solani* [44]. This is consistent with the triggering of the innate immune system by phytoplasma infection, which is consistent with up-regulation of other defence genes, such as the salicylic acid-regulated genes, *PR1*, *PR2* and *PR5*, the jasmonic acid-regulated genes, LOX, AOS, PR3 and PR4, and the ethylene-regulated genes, AP2-like ethylene responsive transcription factor, *Etr* and *ACO,* in grapevines infected with Ca. *P. solani* [43].

In this study, expression of a pear class III chitinase gene was significantly increased in PD-infected leaves, shoots and roots of selection 8824-1, but only in PD-infected roots of selection 9328-1. This suggests that selection 8824-1 has a greater triggering of the innate immune system than selection 9328-1. Expression of a pear PAL gene was significantly increased by PD infection in shoots and roots in both pear selections with neither selection showing a significant difference in leaves. One possibility is that the PAL gene chosen in this study was not related to the innate immune system. PAL gene(s) occur in families in copy numbers up to several dozen in higher plants, and are involved in many plant functions, such as fruit color, flavor and pollinator attraction [45]. The Arabidopsis PAL ortholog that has the most similarity to the pear PAL gene studied in this study is At2g33820, which is primarily involved in structural development and response to oxidative stress and wounding [46]. Examining a pear ortholog of the Arabidopsis PAL gene, such asAt5g04230, perhaps would be more informative as it has been most closely related to the Arabidopsis defence response to pathogens as shown during infection by *Verticillium dahliae* [47].

Expression of a pear ortholog of a phloem protein 2 gene was examined as phloem protein 2 is one of the most common proteins in sieve elements, being involved in sieve cell differentiation, intercellular trafficking by regulating plasmodesmata size, and defence response to limit pathogen colonization by plugging sieve pores [48]. Three phloem 2-like protein genes were up-regulated after symptomatic AP phytoplasma-infected apple trees recovered, based on the disappearance of symptoms [12,49], and a phloem 2-like protein accumulated in citrus plants following infection with the phloem-limited bacterial pathogen, Ca. Liberibacter asiaticus [50]. In the current study, a relatively large significant up-regulation of a pear phloem protein 2 gene was detected in roots of selection 8824-1, which could be limiting PD spread by plugging more phloem sieve elements, thus contributing to lower PD populations in roots of that selection.

While leaf reddening was viewed as a disease symptom of PD infection, it may in fact be part of a host defense response. Anthocyanins are associated with reddening of tissues of *Pyrus* species [51] as well as the innate immune response in plants, such as Arabidopsis, grapevine and poplar [52]. In Arabidopsis, anthocyanin accumulation reduced leaf cell death caused by OY-W phytoplasma compared to mutants defective in anthocyanin biosynthesis [52]. Thus, anthocyanins in the leaf reddening of selection 8824-1 may be part of a stronger triggered innate immunity than selection 9328-1.

## 4. Materials and Methods

### 4.1. Plant Material

Trees of *P. communis* selections 8824-1 and 9328-1 were grown in the AAFC greenhouse facilities at Vineland Station, Ontario, Canada (43°11′29″ N-79°23′48″ W). Buds of the selections were grafted onto 3-year-old *P. communis* rootstocks (Bartlett open-pollinated seedling or clonally-propagated Old Home×Farmingdale 87) in 2006 and the resultant trees were studied 5–6 years later (2011–2012). Tissue samples were collected from both PD-infected (3 trees per each accession) and non-infected (3 trees per each accession) trees in mid-August when the trees started showing foliar symptoms associated with PD. For each sample set, 15–20 randomly-harvested leaves (approx. 40 g) were collected from each tree and then pooled. Also collected from each tree were two pooled shoot tissues per sample set (each 2 cm long internodal tissue) from the mid-section of the tree and one root tissue sample (2 cm length) from approx. 20 cm below the soil level. For shoots and roots, the bark was removed from the cortex to expose the phloem, and then, the phloem was scraped off. The leaf, shoot phloem and root phloem tissues were immediately frozen in liquid nitrogen and stored for up to 2 months at −80 °C until used for RNA or DNA extraction.

### 4.2. Phytoplasma Detection and Quantification

Total DNA was extracted from 500 mg of tissue using a DNeasy Plant Mini Kit (Qiagen, Gaithersburg, MD, USA), and diluted in 100 µL of kit elution buffer. DNA concentrations were measured using a NanoDrop (ND-1000) spectrophotometer (NanoDrop Technologies, Wilmington, DE, USA). For PD phytoplasma quantification, the PD-specific primer pair PHYTOF3/PHYTOR3 (Table 1) [53] was used to amplify a portion of the 16S-23S rRNA intergenic spacer of the PD phytoplasma. PCR reactions were carried out in a 25 μL reaction containing 3 μL of DNA (10 ng/μL), 900 nM PHYTOF3 primer, 450 nM PHYTOR3 primer, 200 mM of TaqMan probe (Table S1) (Integrated DNA Technologies, Coralville, IA, USA), 1.5 mM MgCl_2_ and 1× Taq PCR Master Mix (Qiagen, Gaithersburg, MD, USA) using a Stratagene Model Mx3005P Thermal Cycler (Stratagene, La Jolla, CA, USA). Every sample was run at least in duplicate in the same plate. Reactions were conducted for 5 min at 95 °C, followed by 45 cycles of 15 s at 95 °C and 30 s at 60 °C.

### 4.3. RNA Extraction

Total RNA was extracted from 1.5 g tissue using the Norgen Plant/Fungi RNA Purification kit (Norgen Biotek, Thorold, ON, CA). After extraction, RNA was diluted in 50 µL of elution solution and was treated with RQ1 RNase-free DNase (Promega, Madison, WI, USA) according to the manufacturer’s protocol to remove any DNA from the samples. Concentration and purity of the RNA sample were measured using a NanoDrop ND-1000 spectrophotometer before and after DNase I digestion. Only RNA samples with an OD260/280 ratio between 1.9 and 2.1 and an OD260/230 ratio greater than 2.0 before and after DNase I digestion were used for cDNA synthesis. All RNA samples were diluted to 62.5 ng/µL in nuclease free water.

### 4.4. cDNA Synthesis and Cloning

Complementary DNA (cDNA) was synthesized from RNA in a 20 µL reaction consisting of 8 µL RNA, 4 μL of qScript^TM^ cDNA SuperMix (Quanta BioSciences, Gaithersburg, MD, USA) and 8 µL nuclease free water, and the reaction was conducted as per the manufacturer’s instructions for 5 min at 25 °C, 30 min at 42 °C, 5 min at 85 °C and were held at 4º C in a Techne Cycler TC-512 (Techne, Burlington, NJ, USA). Primers for two carbohydrate metabolism genes (sucrose synthase (*SUSY*) and vacuolar acid invertase 2 (*AIV2*)), four stress/defence-related genes (alcohol dehydrogenase 3 (*ADH3*), chitinase class III (*CHIT.c3*), phenylalanine ammonia lyase (*PAL*), phloem protein 2-3 (*PP2*)) and three constitutive genes (actin (*ACTIN*), glyceraldehyde 3-phosphate dehydrogenase (*GAPDH*) and translation elongation factor-1 alpha (*EF-1α*)), were designed using Primer Express 3.0 software (Applied Biosystems, Foster City, CA, USA) based on sequences obtained from the NCBI nt database (Table 2). PCR conditions were optimized for each gene by gradient PCR to determine annealing temperatures. Reactions contained 10 ng cDNA, 0.25 μM of each primer and 1× Taq PCR Master Mix (Qiagen, Gaithersburg, MD, USA) in a Techne Cycler TC-512 for 2 min at 95 °C, 40 cycles of 30 s at 94 °C, 30 s at 56–60 °C (Table 1), 10 s at 72 °C followed by 5 min at 72 °C and hold at 4 °C.

PCR products were separated on 1.5% TAE agarose gel, purified using a DNA gel extraction kit (Norgen Biotek, Thorold, ON, CA) and cloned into *E. coli* using the pGEM^®®^-T Easy vector system (Promega Corp., Madison, WI, USA). Plasmid DNA was used for standard curves prepared from *E. coli* grown overnight in 3 mL cultures using a plasmid mini-prep kit (Norgen Biotek) and quantified with a NanoDrop ND-1000 spectrophotometer.

### 4.5. Gene Expression and Statistical Analysis

qPCR was conducted in 20 µL reactions containing 3 µL nuclease free water, 10 µL PerfeCTa^®®^ SYBR^®®^ Green FastMix^®®^ Low Rox Reagent (Quanta Biosciences, Gaithersburg, MD, USA), 5 µL 10-fold diluted cDNA and 1 µL each 100 nM forward and reverse primers which were described above for cloning (Table 1). qPCR reactions were performed in a Mx3005P Multiplex Real-time PCR System (Stratagene, La Jolla, CA, USA) with 96-well plates (VWR, West Chester, PA, USA) using 2 min at 95 °C, followed by 45 cycles of 30 s at 95 °C, 30 s at 58–60 °C (Table 1), and 10 s at 72 °C followed by 1 min at 95 °C, 30 s at 55 °C and 30 s at 95 °C. Dissociation curves consisted of 1 min at 95 °C, 30 s at 55 °C and 30 s at 95 °C. Two biological replicates and three technical replicates were used per gene. No template controls were included in all assays to detect non-specific amplification.

Triplicates of 10-fold serial dilutions (10^−2^ to 10^−9^ ng of DNA) of plasmids containing the corresponding cloned insert of the PD phytoplasma 16S-23S rRNA intergenic spacer, pear target genes and pear constitutive reference genes were used to calculate the qPCR efficiency (E) for each primer pair. The calculation was E = 10^−1/slope^, where the slope of the linear regression model fitted over the log-transformed data of the input cDNA concentration versus Ct values according to equation: y = mx + b where y = Ct value, m = slope, x = log_10_ template amount and b = y – intercept [54].

Raw Ct values were transformed to relative quantities using the Mean Normalized Expression formula, $MNE=\frac{{\left(E_{reference}\right)}^{CT\;refrence,mean}}{{\left(E_{target}\right)}^{CT\;target,mean}}$ [55]. Using the MNE method, relative quantities of the 16S-23S rRNA intergenic spacer were determined for phytoplasma quantification in different tissues. Three technical replicates were analyzed for each cDNA sample. Normalized means for the expression of each gene against a chosen constitutive gene were compared using PROC GLM and Tukey’s post hoc test with the SAS statistical software (SAS 9.4; SAS Institute Inc, Cary, NC, USA). A graph for the phytoplasma number was made in R software using ggplot2 and the graphs for gene expression experiments were made with SigmaPlot version 10.0 (Systat Software, Inc., San Jose, CA, USA).

The gene-stability measure (M) was calculated by the NormFinder (an open source algorithm as Microsoft Excel add-in) [56]. Using NormFinder, *GAPDH* was determined to be the reference gene, as it was found to be the gene with the highest stability regardless of the tissue type (Table S1). Detailed methodological procedures are described in the supplementary Methods S1.

## 5. Conclusions

This is the first report showing possible mechanisms by which a phytoplasma infection can result in different symptoms in the same plant species. Symptoms of leaf reddening in selection 8824-1 could be related to it being more tolerant than selection 9328-1 as indicated by lower PD phytoplasma populations in its roots. The tolerance may be expressed as anthocyanin accumulation in leaves and higher expression of genes for alcohol dehydrogenase, class III chitinase and phloem protein 2 following infection. However, the leaf reddening may be a result of premature leaf senescence due to an accumulation of anthocyanins as a result of a build-up of sugars, which would be consistent with the higher sucrose synthase and acid invertase expression in selection 8824-1. In contrast, symptoms of leaf curling may be a reflection of the greater susceptibility of selection 9328-1 with higher PD populations in the roots resulting in greater root malfunction and stress due to decreased water and nutrient transport to leaves. One practical outcome of these findings is that it could improve breeding programs for PD resistance by showing that increased expression of alcohol dehydrogenase, class III chitinase and/or phloem protein 2 might be useful markers in selection for PD phytoplasma resistance, similar to marker-assisted selection strategies applied to pear breeding for other traits [57]. As leaf reddening may be associated with greater resistance, future work could determine whether the effects of PD-induced leaf curling and leaf reddening are similar on yield losses and tree longevity. Future work could also examine expression of a wider range of pear gene expression changes, such as by using RNA-seq analysis as has been done for other phytoplasma diseases [58,59], as well as examining changes in pear physiology associated with symptom development and changes in gene expression.

## Figures and Tables

**Table 1:** Primer and probe sequences and amplicon characteristics for the PD phytoplasma 16S-23S rRNA intergenic spacer for PD quantification (PHTYO), pear housekeeping genes (*ACTIN, EF-1α* and *GAPDH*), pear carbohydrate metabolism genes (*SUSY* and *AIV2*) and pear stress/defence-related genes (*ADH3, PAL, CHIT.c3* and *PP2*).

Name	Primer Sequence [5′–3′]		Amplicon Size (bp)	Annealing Temperature (°C) ^a^	RT-qPCR Efficiency ^b^	R^2 c^
*ACTIN*	F	CCTCCCACATGCCATCCTT	51	60	1.8816	0.99
	R	TCTGTAAGATCACGACCTGCCA				
*GAPDH*	F	CCATATCAAGGGAGGTGCAAA	51	58.4	1.8454	0.99
	R	TCCTTGCTGGGAGCAGAGAT				
*EF-1α*	F	GTGTGATTGAGAGGTTCGAGA	65	58.5	1.8637	0.99
	R	CCAGGCATACTTGAACGACC				
*ADH3*	F	TGTTGGGGAAGGTGTTGAA	42	59.4	1.9413	0.99
	R	CGAGGTTGGTGATCATGT				
*AIV2*	F	ACAATGACCCTCTCCTCACCAAA	63	59.4	1.9931	0.99
	R	GACCAGAATCGGGTTGCCGGAGTA				
*CHIT.c3*	F	GGACAGGCAAAAACGGTCTA	65	58.2	1.9423	0.99
	R	TCTAGGTGAGCATCCGGGAT				
*PAL*	F	GTTGCGCTTTGTCAGTCCGT	60	58.6	1.9658	0.99
	R	TACAGTGTTCCTCAAGTTCTC				
*PP2*	F	CAGCGCAGGATCCAAAAGTC	51	58.5	1.8790	0.99
	R	GGCCGTTGTCATCAGGAATTT				
*SUSY*	F	ATTTCTCAACCGCCACCTTTC	54	58	1.9420	0.98
	R	TCCAACGATTCTCTGTTACGGA				
PHYTOF3/R3 ^d^	F	GTGAATACGTTCTCGGGGTTTGT	136	60	1.9825	0.99
	R	ATACCTTCTTACGACTTAACCCCA				
*TaqMan probe*		FAM-CAATACCCGAAACCAG-IowBlack^®®^ FQ				

^a^ The annealing temperature of the specific PCR product was calculated by using the oligocalc program from oligonucleotide properties calculator (http://biotools.nubic.northwestern.edu/OligoCalc.html; accessed 2 January 2022). ^b^ QPCR efficiency was calculated from the slope(s) of the dilution series standard curve according to the formula E = 10^(−1/slope)^. ^c^ R^2^ is the coefficient of correlation obtained for each standard curve. ^d^ Sequences for primers PHYTOF3/R3 and TaqMan probe were provided by CFIA, Sidney, BC.

**Table 2:** Description of *Pyrus.* sp. housekeeping genes (*ACTIN, EF-1α* and *GAPDH*), carbohydrate metabolism genes (*SUSY* and *AIV2*) and stress/defence-related genes (*ADH3*, *PAL*, *CHIT.c3* and *PP2*) selected for qPCR analysis.

Name	*Pyrus* sp. Homolog ^a^	*Arabidopsis thaliana* Homolog ^b^	*Arabidopsis thaliana* Annotation	Function ^c^	Nt Identity (%) ^d^
*ACTIN*	AF386514	NM_114519	ACTIN-12	structural constituent of cytoskeleton	81
*GAPDH*	AB266449	AK318794	glyceraldehyde-3-phosphate dehydrogenase	glycolysis	100
*EF-1α*	AY338250	AK317216	elongation factor 1-alpha	translation	99
*SUSY*	AB190798	NM_116461	sucrose synthase	carbohydrate metabolism	100
*AIV2*	AB190800	NM_101096	acid beta-fructofuranosidase (vacuolar invertase)	carbohydrate metabolism	100
*ADH3*	AF031899	AY088010	alcohol dehydrogenase 1	stress/defence-related	83
*CHIT.c3*	FJ589785	NM_122314	chitinase A	stress/defence-related	68
*PAL*	DQ230992	NM_129260	Phenylalanine ammonia lyase	stress/defence-related	96
*PP2*	FN395069	NM_102858	phloem protein 2	stress/defence-related	77

^a^ NCBI accession number of nt sequence obtained by searching the GenBank nt database for the corresponding annotation delimited to *Pyrus* species. ^b^ NCBI accession number of the closest *Arabidopsis thaliana* nt sequence identified using the *Pyrus* sp. sequences as a query with a TBLASTX search of the GenBank database. ^c^ Function determined using the gene ontology website: (http://www.geneontology.org/; accessed 2 January 2022). ^d^ Nucleotide identity using BLASTX search of the GenBank nucleotide database with the queries of NCBI accession number of nt sequence.

## Data Availability

The data used in this current study can be available on request to the corresponding author.

## Supplementary Materials

The following are available online at https://www.mdpi.com/article/10.3390/plants11030427/s1, Table S1. NormFinder analysis of the candidate constitutive genes. The NormFinder stability values (M values) were calculated from the Ct values obtained for each cDNA sample for three constitutive genes (ACTIN, GAPDH and EF-1α) in leaf, shoot and root samples in PD phytoplasma infected and non-infected selections 8824-1 and 9328-1. Figure S1. Diagram of the pedigree of *Pyrus communis* selection 8824-1 showing crosses used for generating seed parent. Each column shows one generation. Figure S2. Diagram of the pedigree of *Pyrus communis* selection 9328-1 showing crosses used for generating seed and pollen parents. Each column shows one generation.
