# Supplementary material for: Differences in Gene Expression of Pear Selections Showing Leaf Curling or Leaf Reddening Symptoms Due to Pear Decline Phytoplasma
Source: Plants (Basel). 2022 Feb 4;11(3):427. doi: 10.3390/plants11030427 (PMC8838474; doi:10.3390/plants11030427)
Supplement: Supplementary file 1 [file plants-11-00427-s001.zip › plants-1559200-supplementary.pdf]

Supplementary file 1. Assessment of the stability index of candidate constitutive gene expression

The expression level of each target gene was normalized to the expression level of the validated and most reliable constitutive gene. The constitutive gene should be expressed uniformly in all studied tissues (PD-infected and non-infected leaves, shoots and roots). In this study, actin, glyceraldehyde 3-phosphate dehydrogenase and translation elongation factor-1 alpha were tested for normalizing the expression of the six pear target genes. NormFinder is an algorithm-based software (available at http://moma.dk/) which can be used as a Microsoft Excel Add-in (Andersen *et al*., 2004). NormFinder estimated the variation of constitutive gene expression among different PD-infected and non-infected tissues. Constitutive genes then were ranked based on stability value which is the combined estimation of intra- and intergroup variation of constitutive gene expression. A lower stability value indicates lower variation and more stable expression.

The changes in expression of *ACTIN*, *EF-1α* and *GAPDH* for the three biological replications from individual plant tissues and the combined leaf, shoot and root tissues of the two pear selections were determined. The Ct value for each sample was converted to a linear scale using E^{Ct} equation (E = PCR amplification efficiency) for input into NormFinder (Anderson *et al*., 2004). Log10 transformations of converted data were used by the NormFinder program to analyze the variance of data and to calculate the expected value for each sample. The deviation of the measured value from the expected was used to calculate the stability value (M) that ranked the genes, with the lowest value indicating the most stable one. For selection 8824-1, the gene with the lowest stability value was *GAPDH* with an M value of 0.964 compared to 1.063 for *ACTIN* and 1.529 for *EF-1α* (Table S1). For selection 9328-1, *GAPDH* also had the lowest M value of 0.148 followed by *EF-1α* and actin with 0.180 and 0.428, respectively. For leaves, shoots and roots examined separately, *GAPDH* had the lowest M value in leaves and shoots of selection 8824-1 as well as leaves and roots of selection 9328-1. However, *ACTIN* and *EF-1α* had the lowest M value in roots of selection 8824-1 and shoots of selection 9328-1, respectively. Based on these results, *GAPDH* was chosen as the most stable constitutive control gene for all tissues of selections 8824-1 and 9328-1 and was used for normalizing the qRT-PCR expression data of the six genes for carbohydrate metabolism or stress/defence responses.

Table S1. NormFinder analysis of the candidate constitutive genes. The NormFinder stability values (M values) were calculated from the Ct values obtained for each cDNA sample for three constitutive genes (*ACTIN*, *GAPDH* and *EF-1α*) in leaf, shoot and root samples in PD phytoplasma infected and non-infected selections 8824-1 and 9328-1.

Ranking	Tissue type: Combined Leaf, Shoot and Root		Pear Selection					
			8824-1			9328-1		
			Tissue type			Tissue type		
	8824-1	9328-1	Leaf	Shoot	Root	Leaf	Shoot	Root
**1**	***GAPDH***	***GAPDH***	***GAPDH***	***GAPDH***	***ACTIN***	***GAPDH***	***EF-1α***	***GAPDH***
M value	0.964	0.148	0.015	0.092	0.588	0.002	0.027	0.045
**2**	***ACTIN***	***EF-1α***	***EF-1α***	***EF-1α***	***GAPDH***	***ACTIN***	***GAPDH***	***EF-1α***
M value	1.063	0.180	0.113	0.123	4.952	0.009	0.409	0.638
**3**	***EF-1α***	***ACTIN***	***ACTIN***	***ACTIN***	***EF-1α***	***EF-1α***	***ACTIN***	***ACTIN***
M value	1.529	0.428	0.134	0.923	7.326	0.014	1.236	1.862

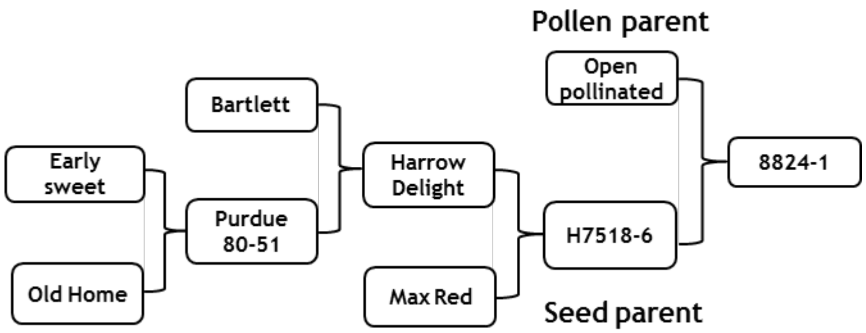

Figure S1. Diagram of the pedigree of *Pyrus communis* selection 8824-1 showing crosses used for generating seed parent. Each column shows one generation.

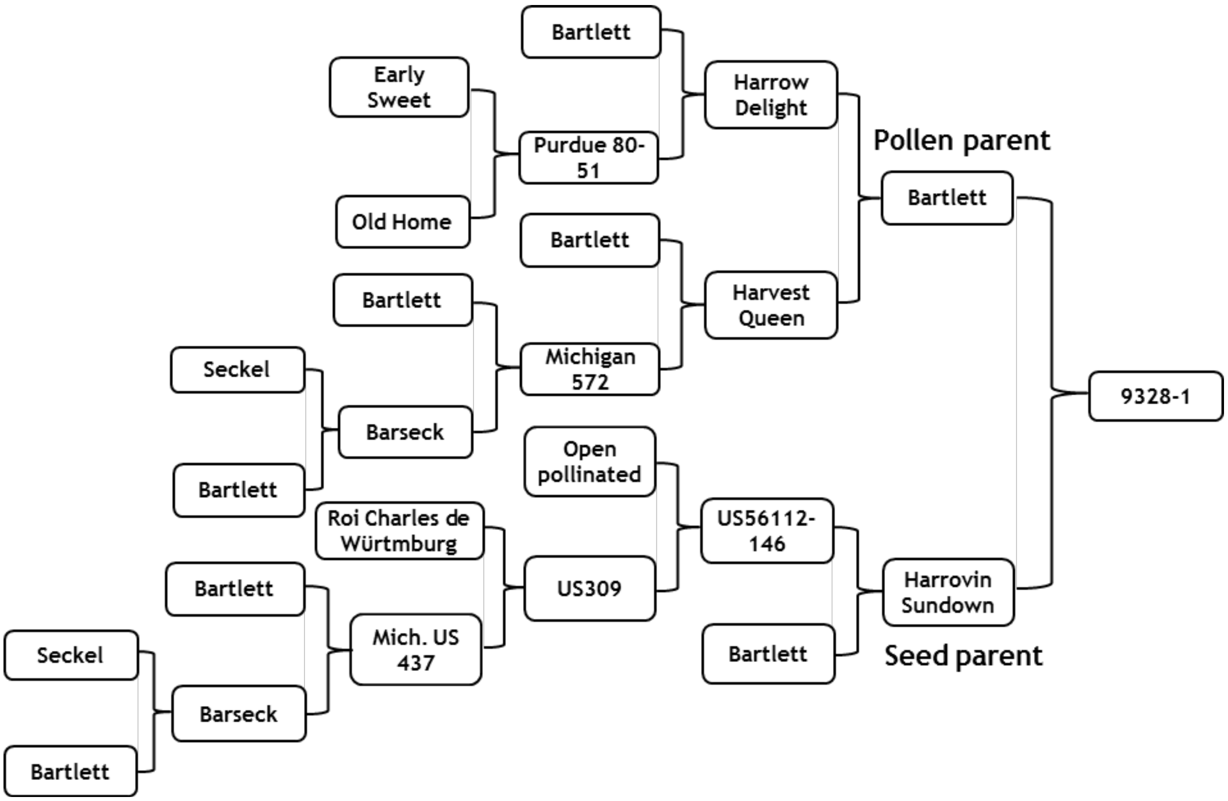

Figure S2. Diagram of the pedigree *of Pyrus communis* selection 9328-1 showing crosses used for generating seed and pollen parents. Each column shows one generation.
